# Supplementary material for: The Physiological Response of the Fiddler Crab Austruca lactea to Anthropogenic Low-Frequency Substrate-Borne Vibrations
Source: Biology (Basel). 2025 Jul 31;14(8):962. doi: 10.3390/biology14080962 (PMC12383940; doi:10.3390/biology14080962)

Supplementary Materials

Figure S1. Map of the crab collection area: (a) the northwestern coastal region of the Republic of Korea showing Yeongjong Island (red box), and (b) the specific collection site.

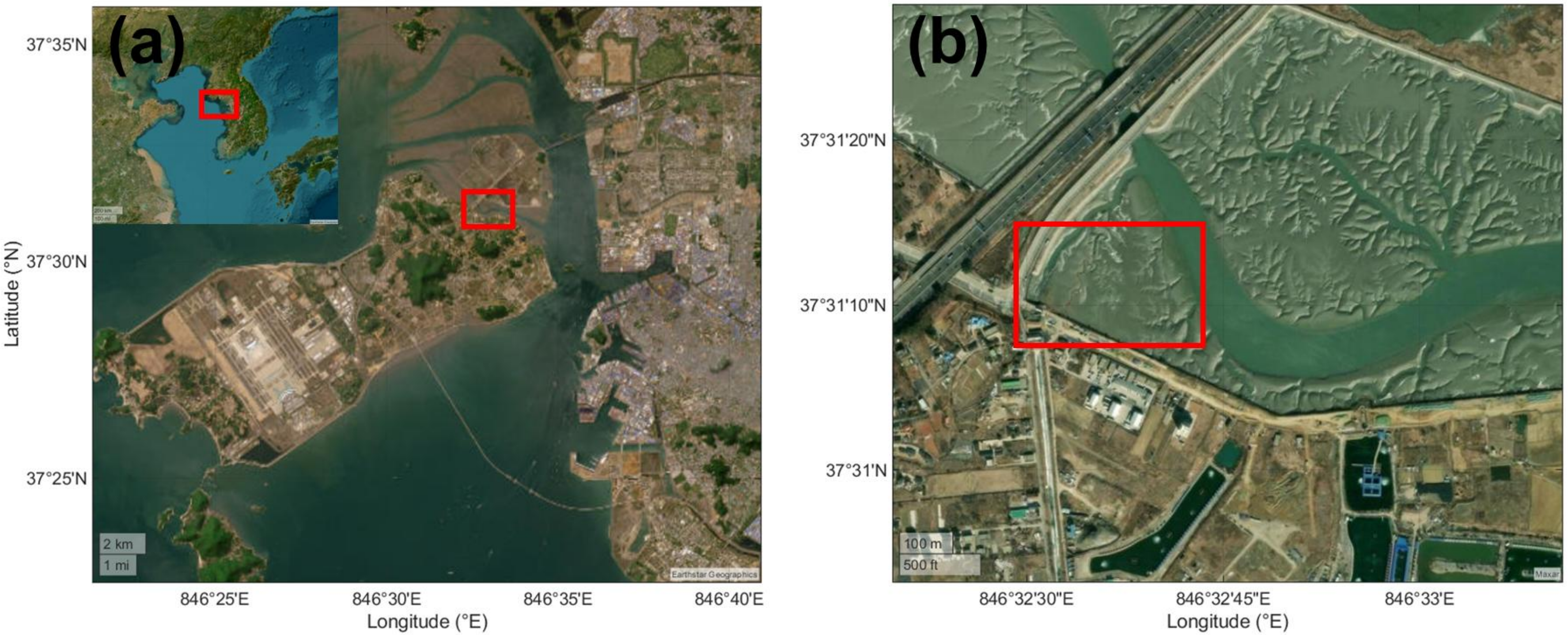

Figure S2. Spectrograms obtained by SFFT analysis of (a) 120 and (b) 250 Hz vibrations.

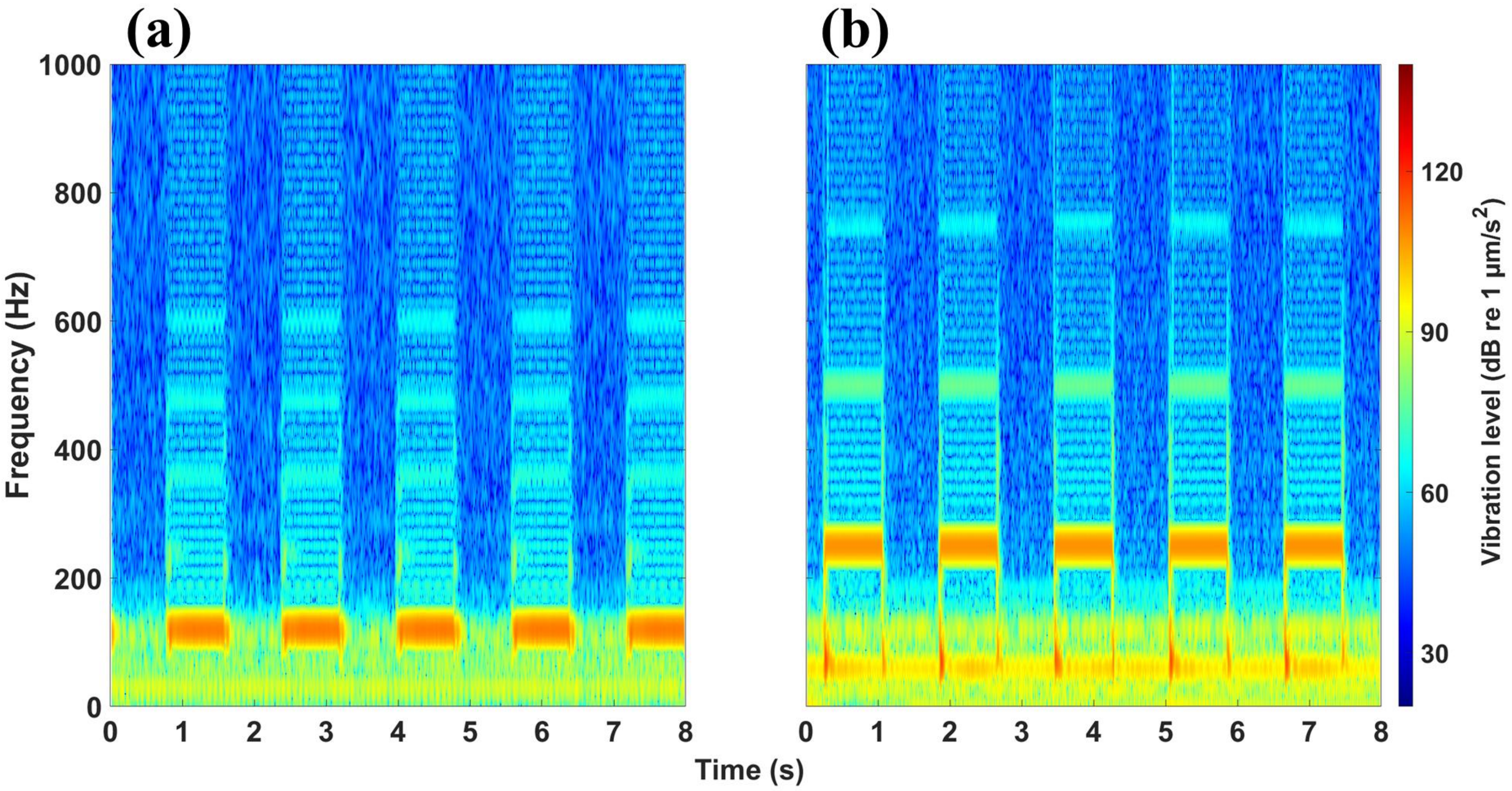

Supplement: Supplementary file 1 [file biology-14-00962-s001.zip › biology-3781696-supplementary.pdf]
